# A Multi-Center Prospective Derivation and Validation of a Clinical Prediction Tool for Severe *Clostridium difficile* Infection

**DOI:** 10.1371/journal.pone.0123405

**Published:** 2015-04-23

**Authors:** Xi Na, Alan J. Martin, Saurabh Sethi, Lorraine Kyne, Kevin W. Garey, Sarah W. Flores, Mary Hu, Dhara N. Shah, Kelsey Shields, Daniel A. Leffler, Ciarán P. Kelly

**Affiliations:** 1 Division of Gastroenterology, Department of Medicine, Beth Israel Deaconess Medical Center and Harvard Medical School, Boston, Massachusetts, United States of America; 2 Department of Medicine for the Older Person, Mater Misericordiae University Hospital and University College Dublin, Dublin, Ireland; 3 University of Houston College of Pharmacy, Houston, Texas, United States of America; Cleveland Clinic, UNITED STATES

## Abstract

**Background and Aims:**

Prediction of severe clinical outcomes in *Clostridium difficile* infection (CDI) is important to inform management decisions for optimum patient care. Currently, treatment recommendations for CDI vary based on disease severity but validated methods to predict severe disease are lacking. The aim of the study was to derive and validate a clinical prediction tool for severe outcomes in CDI.

**Methods:**

A cohort totaling 638 patients with CDI was prospectively studied at three tertiary care clinical sites (Boston, Dublin and Houston). The clinical prediction rule (CPR) was developed by multivariate logistic regression analysis using the Boston cohort and the performance of this model was then evaluated in the combined Houston and Dublin cohorts.

**Results:**

The CPR included the following three binary variables: age ≥ 65 years, peak serum creatinine ≥2 mg/dL and peak peripheral blood leukocyte count of ≥20,000 cells/μL. The *Clostridium difficile* severity score (CDSS) correctly classified 76.5% (95% CI: 70.87-81.31) and 72.5% (95% CI: 67.52-76.91) of patients in the derivation and validation cohorts, respectively. In the validation cohort, CDSS scores of 0, 1, 2 or 3 were associated with severe clinical outcomes of CDI in 4.7%, 13.8%, 33.3% and 40.0% of cases respectively.

**Conclusions:**

We prospectively derived and validated a clinical prediction rule for severe CDI that is simple, reliable and accurate and can be used to identify high-risk patients most likely to benefit from measures to prevent complications of CDI.

## Introduction

*Clostridium difficile* is a gram-positive anaerobic bacterium that is an important human pathogen causing antibiotic associated diarrhea and pseudomembranous colitis [1, 2]. *C*. *difficile* infection (CDI) is highly prevalent in hospitals and nursing homes where patients frequently receive antibiotics and is now a leading cause of hospital-associated infection [1]. The overall incidence of CDI has increased dramatically in the United States, Canada and parts of Europe in recent years [1, 3–5]. Disease severity has also increased with increasingly virulent strains of *C*. *difficile* leading to markedly increased case fatality rates [6]. Nosocomial CDI has been estimated to more than quadruple the cost of otherwise matched hospitalizations, totaling up to $3 billion/year in hospital costs in the United States [7–9]

The clinical outcomes of CDI range from symptomless carriage, to mild diarrhea, to fulminant pseudomembranous colitis and death [1, 10]. Several factors appear to influence the clinical outcome of CDI including the virulence of the infecting strain, the general health of the host and the host immune response [6, 11–13]. There are recent data to indicate that oral vancomycin is superior to metronidazole for treatment of severe CDI [14]. Hence, recent guidelines recommend using oral vancomycin as first-line therapy in patients presenting with severe CDI [1, 15]. These guidelines also acknowledge the need for prospectively validated severity scores for CDI as presented in this study. Thus, it is important to establish clinical prediction tools for severe clinical outcomes in CDI in order to inform early patient management decisions, to improve quality of care to patients suffering from CDI and to facilitate the development and implementation of new interventions to improve clinical outcomes in severe disease. The goal of this study was to develop and validate a simple, easy to apply, clinical prediction rule that could be used at the time of CDI diagnosis to identify patients at high risk for adverse outcomes of the disease.

## Materials and Methods

### Patient Populations and Definitions

CDI was defined as diarrhea, coupled with a positive stool assay for *Clostridium difficile* toxin A or toxin B (evaluated in all cases by enzyme-immunoassay) and not attributed other causes. Diarrhea was defined as a change in bowel habit with 3 or more unformed bowel movements per day for at least 2 days. The primary outcome of interest was “severe CDI” which was defined as a case of CDI leading to any one of the following outcomes: death attributable to CDI or with CDI as a contributing factor; intensive care unit (ICU) admission attributable to CDI or with CDI as a contributing factor; toxic megacolon or colectomy attributable to CDI. The relatedness of ICU admission or death to CDI was determined by chart review performed by two independent physician investigators. In the case of discordant determinations a third physician provided the deciding judgment. All of the severe outcomes evaluated occurred during the same hospital admission when CDI was diagnosed. Our rationale was that severe complications of an acute CDI were likely to occur quite quickly whereas negative outcomes that transpired days or weeks after hospital discharge were unlikely to be related to the index CDI. Patients with CDI were studied at three sites: i) Beth Israel Deaconess Medical Center (BIDMC), Boston, Massachusetts, ii) Mater Misericordiae University Hospital and St Vincent’s University Hospital, Dublin, Ireland and iii) Baylor St. Luke’s Medical Center, Houston, Texas. The data consisted of clinical information and clinical outcomes data for all patients.

### Ethics Statement

The study was approved by the Committee on Clinical Investigations at BIDMC in Boston (local protocol number 2010P-000302), the Committee for the Protection of Research Subjects at the University of Houston (local protocol number CPHS 13101–01), and the Mater Misericordiae University Hospital Ethics Committee (local protocol number 1/378/856). The data presented in this paper from the Houston and Boston sites used information available from patient medical records and as such the protocols at these sites were approved by their respective IRBs with a waiver for informed consent. Written informed consent was obtained from the subjects included in the Dublin cohort as they also participated in a separate larger local study.

### Data collection

Demographic data including age, gender and race as well as relevant laboratory studies including peripheral white blood cell counts and serum creatinine were recorded. Baseline characteristics that were examined included severity of co-morbid conditions (determined by the Charlson comorbidity score), seriousness of underlying illness (determined by the modified Horn disease severity index), immunosuppression (congenital immunodeficiency, HIV or AIDS, active malignancy other than basal or squamous cell skin cancer, immunosuppressive medication post organ transplantation, use of systemic corticosteroids [≥10 mg of oral prednisone or equivalent per day]), chemotherapy agents or other immunosuppressive medications (including anti-TNF antibody therapy, azathioprine, 6-mercaptopurine, cyclosporine or methotrexate within 8 weeks), history of prior CDI, recent use of antibiotics and current acid anti-secretory medications [12, 16–20].

### Clinical Prediction Rule and Statistical Analyses

Initial evaluation of clinical factors associated with severe outcomes of CDI was performed in the Boston cohort. All adult patients with CDI hospitalized at BIDMC between December 2004 and Jan 2006 were eligible for study entry. Factors in the Boston cohort found to be associated with severe CDI on univariate analysis (Table 1) were used to inform a multivariate logistic regression model (Table 2). Evaluation of the data from Dublin and Texas indicated some differences between the three sites in the incidence of severe CDI and in the relative frequency of specific events leading to designation as severe CDI (Table 3). For instance, the Irish cohort had relatively low rates of ICU admission compared to the Boston and Texas sites.

**Table 1:** Characteristics of Boston patients with severe and non-severe CDI (univariate analysis).

Variable	Total cohort	Severe	Non Severe	P value
**Number (%)**	263	51 (19.4%)	212 (80.6%)	NA
**Male gender**	49.8%	51.0%	49.5%	1.0
**Age (years)**	66.5	71.7	65.0	0.018
**Peak WBC (x 10** ^**3**^ **cells/μL)** ^**1**^	15.5	21.3	14.8	0.004
**Baseline creatinine (mg/dL)**	1.77	2.20	1.66	0.15
**Peak creatinine (mg/dL)** ^**1**^	2.40	2.60	1.52	0.048
**GFR nadir (ml/min/1.73m** ^**2**^ **)**	49.8	48.7	51.0	0.014
**Chronic Renal Insufficiency**	17.9%	33.3%	16.0%	0.29
**Immunosuppression**	44.6%	33.3%	46.0%	0.68
**Gastric acid suppression**	75.4%	83.3%	74.5%	0.54
**Ethnicity (% white)**	85.0%	87.8%	84.4%	0.66
**Metronidazole therapy** ^**2**^	93.9%	62.7%	95.2%	0.11
**Charlson score (mean)**	3.28	2.67	3.35	0.50
**Horn’s Index (mean)**	2.25	2.17	2.27	0.41
**History of prior CDI**	15.8%	16.7%	15.7%	1.0

^1^ During the 7 day time period from 5 days before to 2 days after the diagnostic stool sample was obtained.

^2^ Initially treated with metronidazole alone.

**Table 2:** Predictors of severe *C*. *difficle* infection in the Boston cohort (n = 263).

Variable (cut off)	β coefficient	OR	95% CI	Point value assigned
**WBC (≥ 20 x 10** ^**3**^ **cells/μL)**	1.44	4.21	(2.06–8.62)	1
**Creatinine (≥ 2 mg/dL)**	2.09	8.12	(2.51–26.27)	1
**Age (≥ 65 years)**	0.87	2.39	(1.06–5.39)	1

**Table 3:** Characteristics of patients in the Boston, Dublin and Houston cohorts.

Variable	Total cohort	Boston	Dublin	Houston	P values
**Number of patients**	638	263	150	225	NA
**Female gender (%)**					B vs D: 0.15
	53.0%	50.2%	55.3%	52.9%	D vs H: 0.34
					B vs H: 0.59
**Mean age (years) ± SD**					B vs D: 0.07
	67.2 ± 17.1	66.5 ± 17.4	73.5 ± 14.7	63.9 ± 17.1	D vs H: 0.04
					B vs H: 0.53
**Severe CDI (%)**	120 (18.8%)	51 (19.4%)	21 (14.0%)	48 (21.3%)	B vs D^1^: 0.18
***ICU admission***	*65 (10*.*2)%*	*32 (12*.*2)%*	*2 (1*.*3)%*	*31 (13*.*8)%*	D vs H^1^: 0.08
***Megacolon/colectomy***	*12 (1*.*2)%*	*9 (3*.*4)%*	*1 (0*.*7)%*	*2 (0*.*9)%*	B vs H^1^: 0.65
***Death***	*48 (7*.*5)%*	*11 (4*.*2)%*	*19 (12*.*6)%*	*18 (8*.*0)%*	
**Mean peak peripheral WBC (x 10** ^**3**^ **cells/μL)**					B vs D: 0.12
	14.6 ± 10.1	15.5 ± 11.4	15.1 ± 9.1	13.2 ± 9.1	D vs H: 0.72
					B vs H: 0.13
**Mean peak creatinine (mg/dL)**					B vs D: 0.02
	1.8 ± 1.7	1.8 ± 1.8	1.4 ± 1.3	2.0 ± 1.8	D vs H: 0.17
					B vs H: 0.22

^**1**^ Pairwise comparison for total severe CDI.

The Boston cohort was used to derive a clinical prediction rule for severe outcomes of CDI and the combined Dublin and Texas cohorts were used to evaluate the performance of the predication rule. Initial multivariable logistic regression analysis in the derivation cohort suggested age ≥65 years, WBC ≥20,000 cells/μL and serum creatinine ≥2 mg/dl as the main independent variables associated with severe CDI. As the β coefficients for each variable were relatively low (0.87 to 2.09) the presence or absence of each risk factor was made bivariate with one or no points for the presence of each specific risk factor. Risk scores for each patient were then correlated by simple summation of points for all predictors present. For analysis of sensitivity, specificity and positive and negative predictive values, patients were divided into high-risk and low-risk groups based on the presence of two or more risk factors for severe CDI. All statistical analyses were performed using the SPSS software system, version 17.0 (SPSS Inc, Chicago, IL).

## Results

### Patient Characteristics

A total of 638 patients with CDI from three clinical sites were recruited into our study. We prospectively identified 263 consecutive patients with CDI at the Boston site from December 2004 to January 2006 and recorded their clinical information. Similar data were collected prospectively for 150 patients with CDI at the Dublin site from November 2007 to June 2009 and for 225 subjects with CDI at the Houston site from January 2006 to August 2010. Forty two subjects lacked essential clinical data leaving a total of 596 evaluable subjects. Overall 53.0% of subjects were female; gender distribution was similar at all three sites (Table 3). The patients from Dublin and Houston were similar with respect to age while the subjects from Dublin were slightly older (Table 3). A total of 120 patients (18.8%) met the criteria for severe clinical outcomes of CDI (Table 3); 51 out of 263 (19.4%) patients from Boston, 21 out of 150 (14.0%) patients from Dublin and 48 out of 225 (21.3%) subjects from Houston. ICU admission was less frequent in the Dublin patients (1.3%) compared to Boston (12.2%) and Houston (13.8%). Megacolon/colectomy was more frequent in the Boston patient (3.4%) compared to Dublin (0.7%) and Houston (0.9%) patients. Death related to CDI occurred in 4.2% of the patients in Boston compared to 6.7% of the patients in Dublin and 8.0% of the patients in Houston.

### Development of a Clinical Prediction Rule for Severe CDI

We first examined patient characteristics and clinical data for the 263 CDI patients in Boston. Univariate analysis identified age, peak peripheral blood white blood cells (WBC) (evaluated within the time period from 5 days before to 2 days after the diagnostic stool sample was obtained) and peak serum creatinine (evaluated within the same 7 day time period) as being significantly higher in CDI patients with severe disease when compared to the non-severe CDI group (Table 1). The nadir of the calculated glomerular filtration rate was also significantly associated with severe CDI but the final clinical prediction rule was not substantially improved by using GFR and so the more simple measure of serum creatinine was used in preference. The other variables examined were not significantly associated with severe CDI. Based on these findings we generated a clinical prediction model for severe CDI outcomes as shown in Table 2. The β coefficients for each of the three predictors (age ≥65 years, peak serum creatinine ≥ 2 mg/dl and peak WBC ≥ 20 x 10^3^ cells/μL) were similar and so each was assigned a score value of one.

**Fig 1:**
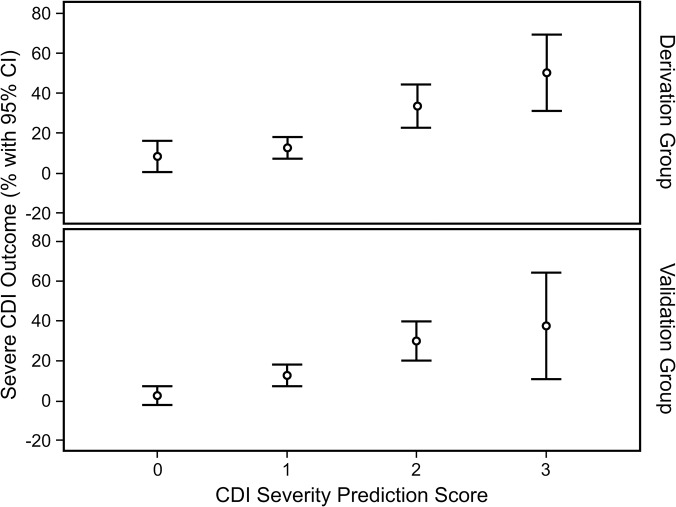
Association between CDI severity prediction score (0 to 3) and severe outcomes of CDI in the derivation (upper panel) and validation (lower panel) groups.

As illustrated in Fig 1, upper panel, in the derivation group, patients with higher CDI severity scores showed a greater probability to develop severe clinical outcomes of CDI. Individuals’ scores of 0, 1, 2 or 3 had a risk of severe clinical outcomes of CDI of 7.1%, 12.1%, 40.7% and 58.3% respectively (Table 4).

**Table 4:** Risk of severe outcomes of *C*. *difficile* infection according to Prediction Score^1^.

	Derivation Cohort (Boston)				Validation Cohort (Dublin+Houston)			
Score	Severe	Non-Severe	Total	% Severe	Severe	Non-Severe	Total	% Severe
0	4	52	56	7.1%	2	41	43	4.7%
1	15	109	124	12.1%	28	175	203	13.8%
2	24	35	59	40.7%	28	56	84	33.3%
3	7	5	12	58.3%	6	9	15	40.0%
0 or 1	19	161	180	10.5%	30	216	246	12.2%
2 or 3	31	40	71	43.7%	34	65	99	34.3%

^1^ Of the 638 subjects included in this study, 42 lacked essential clinical data. The total number of evaluable subjects included in this table is 596.

Using a dichotomized score, patients in the derivation group with a low CDI severity score (0 or 1) had a 10.5% risk of severe outcomes as compared to 43.7% in those with a high severity score (2 or 3). The sensitivity, specificity, positive predictive value and negative predictive value of a high CDI severity score (2 or 3) are presented in Table 5. The overall diagnostic accuracy of a high (2 or 3) or a low (0 or 1) CDI severity score in predicting severe versus non-severe CDI clinical outcomes was 76.5% in the derivation group.

**Table 5:** Performance Clinical Prediction Score for Severe *C*. *difficile* Infection.

	Derivation cohort (n = 251)	Validation cohort (n = 345)
**Severe CDI**	12.3% (31/251)	9.9% (34/345)
**Sensitivity**	62% (31/50; 95% CI: 48.15–74.14)	53.1% (34/64; 95% CI: 41.08–64.83)
**Specificity**	80.1% (161/201; 95% CI: 74.04–85.03)	76.9% (216/281; 95% CI: 71.60–81.42)
**PPV**	43.7% (31/71; 95% CI: 32.74–55.23)	34.3% (34/99; 95% CI: 25.73–44.12)
**NPV**	89.4% (161/180; 95% CI: 84.10–93.13)	87.8% (216/246; 95% CI: 83.12–91.32)
**Overall predictive accuracy**	76.5% (192/251; 95% CI: 70.87–81.31)	72.5%, (250/345; 95% CI: 67.52–76.91)
**Positive Likelihood Ratio**	3.12 (95% CI: 2.19–4.43)	2.30 (95% CI: 1.1968–3.14)
**Negative Likelihood Ratio**	0.47 (95% CI: 0.33–0.68)	0.61 (95% CI: 0.47–0.80)

### Validation of the Clinical Prediction Rule

In order to perform a formal validation of the clinical prediction rule for risk of severe clinical outcomes in CDI, we used combined Houston and Dublin cohorts as the validation cohort.

When the prediction rule was applied to the validation cohort the correlation between the prediction score and the clinical outcome of severe CDI was again clearly evident (Fig 1, lower panel). In the validation group, individuals’ risk prediction scores of 0, 1, 2 or 3 were associated with severe clinical outcomes of CDI in 4.7%, 13.8%, 33.3% and 40.0% of cases respectively (Table 4).

Using a dichotomized score, patients in the validation group with a low CDI severity score (0 or 1) had a 12.2% risk of severe outcomes as compared to 34.3% in those with a high severity score (2 or 3). The sensitivity, specificity, positive predictive value, negative predictive value, positive likelihood ratio and negative likelihood ratio of a high CDI severity score (2 or 3) are presented in Table 5. The overall diagnostic accuracy of a high (2 or 3) or a low (0 or 1) CDI severity score in predicting severe versus non-severe CDI clinical outcomes was 72.5% in the validation group.

### The Performance of the Severe CDI Risk Score

**Fig 2:**
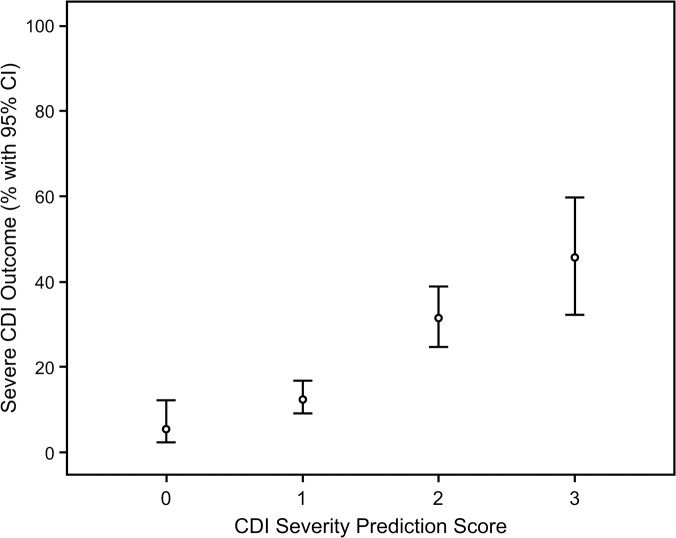
Association between CDI severity prediction score (0 to 3) and severe outcomes of CDI in the entire cohort (n = 596).

When sorted by risk scores, the prediction score identified groups of patients with increasing probability of severe outcomes of CDI as illustrated in Fig 2. In the entire cohort of 596 individuals those with a CDI severity score of 0 had a risk of severe clinical outcomes of 6.7%, individuals with a score of 1 had a risk of severe clinical outcomes of 16.3%, those with a score of 2 had a risk of severe clinical outcomes of 34.9% and a score of 3 was associated with a 46.9% risk of severe clinical outcomes of CDI. Using a dichotomized scoring system of lower (0 or 1) versus higher risk (2 or 3) the incidence of severe CDI clinical outcomes were 11.5% and 38.2% respectively.

**Fig 3:**
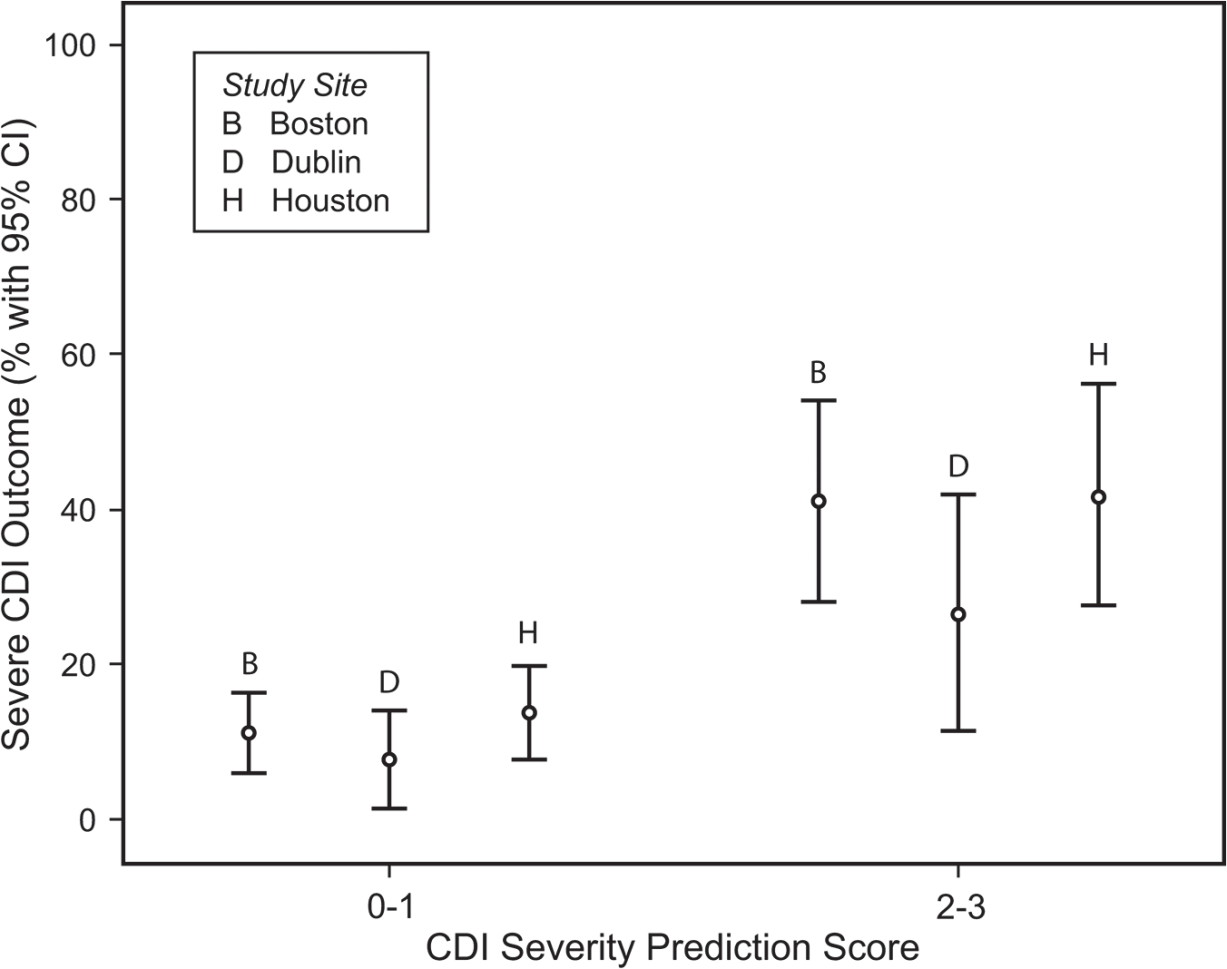
Association between CDI severity prediction score (0–1 or 2–3) and severe outcomes of CDI at each of the three study sites (B = Boston, D = Dublin, H = Houston).

When the CDI severity score was applied to the patients according to their study site those with higher scores (2 or 3) were at substantially greater risk for severe clinical outcomes from CDI when compared to those with lower scores (0 or 1) at each of the three sites (Fig 3). Patients with a low CDI severity score of 0 or 1 had a risk of severe CDI of 10.6% in Boston, 7.9% in Dublin and 14.6% in Houston. Conversely, patients with a CDI severity risk score of 2 or 3 had a higher probability of developing severe CDI at each site: 43.7% in Boston, 25% in Dublin and 40.7% in Houston (Fig 3).

## Discussion

*Clostridium difficile* infection (CDI) is a common cause of nosocomial diarrhea that is associated with substantial morbidity, mortality and healthcare costs. First-line antibiotic treatment for mild or moderately severe CDI is metronidazole. However, oral vancomycin is recommended for severe CDI. Some patients require intensive care unit admission, and colectomy is necessary for others [1, 21]. Mortality rates from CDI in the United States increased from 5.7 per million population in 1999 to 23.7 per million in 2004 [22]. Several new therapeutic agents are being developed for CDI. Fidaxomicin is a new, macrocyclic antibiotic, recently approved for use in CDI and is associated with lower rates of recurrent disease as compared to oral vancomycin [23, 24]. Monoclonal antibodies directed against *C*. *difficile* toxins have also been found to be effective in preventing CDI recurrences [25]. It is becoming increasingly important to identify patients with a greater likelihood for severe disease who would be most likely to benefit from receiving more aggressive treatment with vancomycin, or possibly novel therapies, early on in their clinical course.

Clinical prediction rules (CPRs) can help to address these aforementioned challenges in CDI management and, accordingly, several CPRs for CDI complications have been proposed over the years; but none has gained widespread clinical acceptance. A systematic review published in 2012 that specifically addressed CPRs for poor outcomes in CDI concluded that the available CPRs lack weighing variables and strong validation thereby rendering their quality suboptimal and utility debatable [26]. As the authors state: “Evidence-based tools developed through appropriate prospective cohorts would be more valuable for clinicians than empirically-developed CPRs” [26]. The current study addressed these limitations and as such is positioned to become a valuable addition to the field.

In this study we prospectively identified, using multivariate analysis, risk factors identified at the time of CDI diagnosis that independently predict later severe clinical outcomes, or complications, of the disease. Using these risk factors we developed a *Clostridium difficile* severity score (CDSS) that uses readily available clinical data to predict severe outcomes of CDI. We then validated the CDSS in a separate group of CDI patients. Our study is, to our knowledge, the largest prospective, multi-center study to develop and validate a clinical prediction rule for severe clinical outcomes of CDI reported to date. A total of 638 CDI patients from three institutions were included in our study. Objective markers for severe complications of CDI were used (colectomy, admission to an intensive care unit or death from CDI or with CDI as a contributor). Our study indicates that three simple clinical criteria (age greater than 65 years, peak serum creatinine greater than 2 mg/dL and peak peripheral blood leukocyte count ≥ 20,000 cells/μL) can be used to identify patients at greatest risk for severe CDI complications. At initial diagnosis, most patients with CDI present with similar symptoms that do not correlate with the subsequent clinical course of their disease. The CDSS can be determined easily at the time of diagnosis of CDI and alert clinicians to a greater risk for serious complications indicating the need for more aggressive management e.g. use of oral vancomycin as the first-line agent and discontinuing all other non-essential antimicrobial drugs. The CDSS can also be valuable in developing, testing and applying new treatment approaches to avert severe complications of CDI.

A recent systematic review identified 13 studies on the derivation of a CPR for CDI: two on recurrences, five on complications, five on mortality and one on response to treatment [26]. The authors noted that there were a number of limitations in these studies i.e. heterogeneity in the variables used, small sample size, and only a few studies performed multivariate analyses to adjust for confounders. In that review, the authors found that only three studies (Lungulescu et al., Bhangu et al. and Zilberberg et al.) dealing with CDI severity had a good quality of derivation methodology [27–29].

Lungulescu et al. created a CDI severity index (CSI) score based on four risk factors that were identified by univariate analysis: history of malignancy, white blood cell count at admission ≥ 20,000/dL, blood albumin <3.0mg/dL and creatinine at admission >1.5 fold the baseline value. One point was assigned to each of the risk factors. A CSI score with a cut-off value of 2 had a sensitivity and specificity of 82% and 65%, respectively. Our study reaffirms the clinical significance of having an elevated leukocyte count and creatinine at the time of CDI diagnosis in predicting severe CDI. In our study data were collected prospectively—in keeping with the general principal that predictive variables should be collected prospectively—and therefore more accurately than in prior studies, in a process established specifically for the development and the validation of clinical rules [30, 31]. The study by Lungulescu et al. is also limited by not using multivariate analyses and lacking a validation cohort.

Bhangu et al. created a score based on five risk factors that were identified by a multi-variate analysis: age ≥ 80 years, clinically severe disease, white blood cell count ≥ 20,000/dL or CRP ≥150 mg/dL, blood urea nitrogen > 15 mmol/L and albumin < = 2.0mg/dL. One point was assigned to each of the risk factors. Point counts of 0–1, 2–3 and 4–5 were associated with mortality rates of 22%, 55% and 89% respectively. Bhangu et al. collected the data prospectively. However, they did not have a validation cohort and did not report performance parameters of scores.

Zilberberg et al. created a score based on five risk factors that were identified by a multi-variate analysis: age ≥ 75 y, no respiratory disease, septic shock, lack of leukocytosis, APACHE II score ≥20. Absence of chronic respiratory disease (R), age 75+ yrs (A), septic shock (S) and Acute Physiology and Chronic Health Evaluation II score 20+ (A) comprised the RASA score, whose receiver operating characteristics was 0.740; 95% Confidence Interval was 0.663–0.817. The RASA score was put forth as a prediction tool for 30-day mortality and clinical management in an elderly population afflicted by CDI. Older age was one of the variables in Zilberberg’s score initially derived to predict mortality among the elderly, but the weight given to age was potentially over-estimated by being also included within the APACHE II score. Other limitations of the study include retrospective data collection and the absence of a validation cohort.

Horn's index, a severity score based on underlying clinical illness, has also been shown to be to be a useful method for identifying CDI patients at high risk of poor clinical and economic outcomes [20]. A recent prospective study from Texas demonstrated that patients with Horn's index scores of 3 or 4 had a significantly longer hospital stay (mean 33.4 [standard deviation, SD 33.3] days) than patients with scores of 1 or 2 (mean 15.1 [SD 16.2] days, P = 0.001) [12]. The study was smaller (85 subjects) and validation was not performed. Also, Horn’s index is a subjective assessment by a physician; it is not known whether clinicians from different regions would assign the same Horn’s score to a specific patient.

Although our current model addresses several weaknesses of previous studies there are also some methodological limitations. We recognize that dichotomizing continuous variables sacrifices information, but we opted to do so to increase the ease of clinical application of our prediction tool. It would be ideal to validate this model in a large external observational cohort, but unfortunately, we did not have access to data from another prospective cohort study of CDI of similar size. Therefore, we opted to conduct internal validation using the split-sample method. The predictive model we developed and validated is consistent with the risk factors identified in previous studies and updating or validating the model externally would involve minor calibration, if any. We believe that the risk score we developed is generalizable to other CDI cohorts. Indeed, our model worked well despite differences in the healthcare systems, study populations and the frequency of severe outcomes among the Boston, Houston and Ireland cohorts included in our analysis. Another potential limitation is that we did not perform strain typing of *C*. *difficile* isolates in this study. Some, but not all, studies have found that infection with specific strains of *C*. *difficile* (such as ribotypes 027, 078 or 244) may be associated with more severe outcomes.[5, 6, 18, 32] In previous studies 25% of isolates from hospital patients at the Boston site were ribotype 027 (BI/NAP-1), 24% were ribotype 027 and 6% were 078 in Houston whereas in Dublin 26% were 027 and 14% were 078. Ribotype 244 was not identified.[18, 33] However, strain type information is rarely available to clinicians at the time of diagnosis and treatment of *C*. *difficile* infection. Hence, we did not consider it a practical or valuable inclusion for a clinical prediction tool.

In summary, we derived and validated a predication rule for severe CDI and showed that the risk of severe CDI can be reliably predicted using a simple risk-scoring system. Consistent with prior studies, advanced age, elevated leukocytes and creatinine were associated with severe clinical outcomes of CDI. This score can be helpful to practitioners to identify patients at high risk of developing severe CDI outcomes and to provide aggressive treatment with vancomycin early on in their clinical course. The score will also help in risk stratification for targeting novel interventions appropriately. Further, prospective validation will be important to consolidate and refine the prediction rule.

## References

[1] Cohen SH, Gerding DN, Johnson S, Kelly CP, Loo VG, McDonald LC, et al Clinical practice guidelines for Clostridium difficile infection in adults: 2010 update by the society for healthcare epidemiology of America (SHEA) and the infectious diseases society of America (IDSA). Infect Control Hosp Epidemiol. 2010;31(5):431–55. 10.1086/651706 20307191

[2] Kelly CP, LaMont JT. Clostridium difficile—more difficult than ever. N Engl J Med. 2008;359(18):1932–40. 10.1056/NEJMra0707500 18971494

[3] Dubberke ER, Butler AM, Yokoe DS, Mayer J, Hota B, Mangino JE, et al Multicenter study of Clostridium difficile infection rates from 2000 to 2006. Infect Control Hosp Epidemiol. 2010;31(10):1030–7. 10.1086/656245 20695799 PMC3648217

[4] McDonald LC, Owings M, Jernigan DB. Clostridium difficile infection in patients discharged from US short-stay hospitals, 1996–2003. Emerg Infect Dis. 2006;12(3):409–15. 16704777 10.3201/eid1203.051064 PMC3291455

[5] Loo VG, Poirier L, Miller MA, Oughton M, Libman MD, Michaud S, et al A predominantly clonal multi-institutional outbreak of Clostridium difficile-associated diarrhea with high morbidity and mortality. N Engl J Med. 2005;353(23):2442–9. 16322602 10.1056/NEJMoa051639

[6] McDonald LC, Killgore GE, Thompson A, Owens RC Jr., Kazakova SV, Sambol SP, et al An epidemic, toxin gene-variant strain of Clostridium difficile. N Engl J Med. 2005;353(23):2433–41. 16322603 10.1056/NEJMoa051590

[7] Dubberke ER, Reske KA, Olsen MA, McDonald LC, Fraser VJ. Short- and long-term attributable costs of Clostridium difficile-associated disease in nonsurgical inpatients. Clin Infect Dis. 2008;46(4):497–504. 10.1086/526530 18197759

[8] Kyne L, Hamel MB, Polavaram R, Kelly CP. Health care costs and mortality associated with nosocomial diarrhea due to Clostridium difficile. Clin Infect Dis. 2002;34(3):346–53. 11774082 10.1086/338260

[9] O’Brien JA, Lahue BJ, Caro M J Jaime, Davidson DM. The emerging infectious challenge of Clostridium difficile–associated disease in Massachusetts hospitals: clinical and economic consequences. Infect Control Hosp Epidemiol. 2007;28(11):1219–27. 17926270 10.1086/522676

[10] Kelly CP. A 76-year-old man with recurrent Clostridium difficile-associated diarrhea: review of C. difficile infection. JAMA. 2009;301(9):954–62. 10.1001/jama.2009.171 19190304

[11] Wilson V, Cheek L, Satta G, Walker-Bone K, Cubbon M, Citron D, et al Predictors of death after Clostridium difficile infection: a report on 128 strain-typed cases from a teaching hospital in the United Kingdom. Clin Infect Dis. 2010;50(12):e77–81. 10.1086/653012 20450417

[12] Arora V, Kachroo S, Ghantoji SS, Dupont HL, Garey KW. High Horn's index score predicts poor outcomes in patients with Clostridium difficile infection. J Hosp Infect. 2011;79(1):23–6. 10.1016/j.jhin.2011.04.027 21700363

[13] Kelly CP, Kyne L. The host immune response to Clostridium difficile. J Med Microbiol. 2011;60(Pt 8):1070–9. 21415200 10.1099/jmm.0.030015-0

[14] Zar FA, Bakkanagari SR, Moorthi KM, Davis MB. A comparison of vancomycin and metronidazole for the treatment of Clostridium difficile-associated diarrhea, stratified by disease severity. Clin Infect Dis. 2007;45(3):302–7. 17599306 10.1086/519265

[15] Bauer MP, Kuijper EJ, van Dissel JT. European Society of Clinical Microbiology and Infectious Diseases (ESCMID): treatment guidance document for Clostridium difficile infection (CDI). Clin Microbiol Infect. 2009;15(12):1067–79. 10.1111/j.1469-0691.2009.03099.x 19929973

[16] Hu MY, Katchar K, Kyne L, Maroo S, Tummala S, Dreisbach V, et al Prospective derivation and validation of a clinical prediction rule for recurrent Clostridium difficile infection. Gastroenterology. 2009;136(4):1206–14. 10.1053/j.gastro.2008.12.038 19162027

[17] Hu MY, Maroo S, Kyne L, Cloud J, Tummala S, Katchar K, et al A prospective study of risk factors and historical trends in metronidazole failure for Clostridium difficile infection. Clin Gastroenterol Hepatol. 2008;6(12):1354–60. 10.1016/j.cgh.2008.06.024 19081526 PMC2644212

[18] Cloud J, Noddin L, Pressman A, Hu M, Kelly C. Clostridium Difficile Strain NAP-1 Is Not Associated With Severe Disease In a Nonepidemic Setting. Clin Gastroenterol Hepatol. 2009. 10.1016/j.cgh.2009.05.018 19465153

[19] Charlson ME, Pompei P, Ales KL, MacKenzie CR. A new method of classifying prognostic comorbidity in longitudinal studies: development and validation. J Chronic Dis. 1987;40(5):373–83. 3558716 10.1016/0021-9681(87)90171-8

[20] Horn SD, Sharkey PD, Bertram DA. Measuring severity of illness: homogeneous case mix groups. Med Care. 1983;21(1):14–30. 6403781 10.1097/00005650-198301000-00002

[21] McDonald L, Coignard B, Dubberke E, Song X, Horan T, Kutty PK, et al Recommendations for surveillance of Clostridium difficile–associated disease. Infect Control Hosp Epidemiol. 2007;28(2):140–5. 17265394 10.1086/511798

[22] Redelings MD, Sorvillo F, Mascola L. Increase in Clostridium difficile–related mortality rates, United States, 1999–2004. Emerg Infect Dis. 2007;13(9):1417 10.3201/eid1309.061116 18252127 PMC2857309

[23] Cornely OA, Crook DW, Esposito R, Poirier A, Somero MS, Weiss K, et al Fidaxomicin versus vancomycin for infection with Clostridium difficile in Europe, Canada, and the USA: a double-blind, non-inferiority, randomised controlled trial. Lancet Infect Dis. 2012;12(4):281–9. 10.1016/S1473-3099(11)70374-7 22321770

[24] Louie TJ, Miller MA, Mullane KM, Weiss K, Lentnek A, Golan Y, et al Fidaxomicin versus vancomycin for Clostridium difficile infection. N Engl J Med. 2011;364(5):422–31. 10.1056/NEJMoa0910812 21288078

[25] Lowy I, Molrine DC, Leav BA, Blair BM, Baxter R, Gerding DN, et al Treatment with monoclonal antibodies against Clostridium difficile toxins. N Engl J Med. 2010;362(3):197 10.1056/NEJMoa0907635 20089970

[26] Abou Chakra CN, Pepin J, Valiquette L. Prediction tools for unfavourable outcomes in Clostridium difficile infection: a systematic review. PLoS One. 2012;7(1):e30258 10.1371/journal.pone.0030258 22291926 PMC3265469

[27] Bhangu S, Bhangu A, Nightingale P, Michael A. Mortality and risk stratification in patients with Clostridium difficile‐associated diarrhoea. Colorectal Dis. 2010;12(3):241–6. 10.1111/j.1463-1318.2009.01832.x 19508548

[28] Lungulescu O, Cao W, Gatskevich E, Tlhabano L, Stratidis J. CSI: a severity index for Clostridium difficile infection at the time of admission. J Hosp Infect. 2011;79(2):151–4. 10.1016/j.jhin.2011.04.017 21849220

[29] Zilberberg MD, Shorr AF, Micek ST, Doherty JA, Kollef MH. Clostridium difficile-associated disease and mortality among the elderly critically ill. Crit Care Med. 2009;37(9):2583–9. 10.1097/CCM.0b013e3181ab8388 19623053

[30] Laupacis A, Sekar N. Clinical prediction rules: a review and suggested modifications of methodological standards. JAMA. 1997;277(6):488–94. 9020274

[31] May S, Rosedale R. Prescriptive clinical prediction rules in back pain research: a systematic review. Journal of Manual & Manipulative Therapy. 2009;17(1):36–45. 20046564 10.1179/106698109790818214 PMC2704349

[32] Pepin J, Valiquette L, Gagnon S, Routhier S, Brazeau I. Outcomes of Clostridium difficile-associated disease treated with metronidazole or vancomycin before and after the emergence of NAP1/027. Am J Gastroenterol. 2007;102(12):2781–8. 17900327 10.1111/j.1572-0241.2007.01539.x

[33] Burns K, Skally M, Solomon K, Scott L, McDermott S, O'Flanagan D, et al Clostridium difficile infection in the Republic of Ireland: results of a 1-month national surveillance and ribotyping project, March 2009. Infect Control Hosp Epidemiol. 2010;31(10):1085–7. 10.1086/656376 20731596

